# Association of mannose-binding lectin 2 gene polymorphisms with Guillain-Barré syndrome

**DOI:** 10.1038/s41598-022-09621-y

**Published:** 2022-04-06

**Authors:** Israt Jahan, Shoma Hayat, Mir M. Khalid, Rijwan U. Ahammad, Asaduzzaman Asad, Badrul Islam, Quazi D. Mohammad, Bart C. Jacobs, Zhahirul Islam

**Affiliations:** 1 Laboratory of Gut-Brain Signaling, Laboratory Sciences and Services Division, icddr, b, Mohakhali, Dhaka, 1212 Bangladesh; 2 Department of Medical Microbiology and Infectious Diseases, Erasmus MC, University Medical Center, Rotterdam, The Netherlands; 3 Graduate School of Medicine, Nagoya University, Nagoya, Japan; 4 National Institute of Neurosciences and Hospital, Dhaka, Bangladesh; 5 Department of Neurology and Immunology, Erasmus MC, University Medical Center, Rotterdam, The Netherlands

**Keywords:** Molecular biology, Biomarkers, Neurology, Pathogenesis

## Abstract

Complement activation plays a critical role in the pathogenesis of Guillain-Barré syndrome (GBS), a debilitating immune-mediated neuropathy. Mannose-binding lectin (MBL) is a complement activation factor of lectin pathway which as genetic host factor may influence the susceptibility or severity of GBS. We investigated the frequency of *MBL2* promoter (− 550H/L and − 221X/Y) and functional region (exon 1 A/O) polymorphisms and their association with disease susceptibility, clinical features and serum MBL among GBS patients (*n* = 300) and healthy controls (*n* = 300) in Bangladesh. The median patient age was 30 years (IQR: 18–42; males, 68%). *MBL2* polymorphisms were not significantly associated with GBS susceptibility compared to healthy controls. HL heterozygosity in GBS patients was significantly associated with mild functional disability at enrolment (*P* = 0.0145, OR, 95% CI 2.1, 1.17–3.82). The HY, YA, HA and HYA heterozygous haplotypes were more common among mildly affected (*P* = 0.0067, *P* = 0.0086, *P* = 0.0075, *P* = 0.0032, respectively) than severely affected patients with GBS. Reduced serum MBL was significantly associated with the LL, OO and no HYA variants and GBS disease severity. No significant association was observed between *MBL2* polymorphisms and electrophysiological variants, recent *Campylobacter jejuni* infection or anti-ganglioside (GM1) antibody responses in GBS. In conclusion, *MBL2* gene polymorphisms are related to reduced serum MBL and associated with the severity of GBS.

## Introduction

Guillain-Barré syndrome (GBS) is a rapidly progressing peripheral neuropathy and the major cause of flaccid paralysis after an acute infection^1^. The global incidence of GBS is 1–2 per 100,000 person/year, though this rate varies regionally with significant heterogeneity in terms of clinical presentation and severity^2,3^. Two-third of patients have symptoms of a precedent infection^4^ and the most identified cause is *Campylobacter jejuni*^5^. The recent public health outbreak of Zika virus caused many patients to develop neurological symptoms like GBS^6^. However, the mechanisms underlying GBS have not yet been elucidated. There are two major variants of GBS: axonal (AMAN; acute motor axonal neuropathy and AMSAN; acute motor-sensory axonal neuropathy) and demyelinating (AIDP; acute inflammatory demyelinating polyradiculoneuropathy)^1^. Conclusive evidence indicates GBS after *C. jejuni* infection is an auto-antibody mediated disease triggered by molecular mimicry between the surface epitopes of an infection agent and peripheral nerve gangliosides, which accounts for the pathogenesis of axonal GBS^7^. Very few patients with a microbial infection develop GBS^8^, which indicates involvement of host factors triggering this autoimmunity. Genetic susceptibility could be a predisposing factor for GBS, though the genetic factors that affect the interactions between microbial and host are poorly studied.

The host complement system plays a crucial role in the pathogenetic mechanism of GBS; by mediating complement fixations through anti-ganglioside antibody that elevated serum complements and deposited on the surface of Schwann cells and nodes of Ranvier^9,10^. Over the last decade, mannose-binding lectin (MBL) has been the center of substantial interest as it possesses the ability to turn on the complement pathway through enzymatic regulation and thus may influence disease susceptibility and severity^11^. MBL is encoded by the *MBL2* gene, which contains four exons. Three single nucleotide polymorphisms (SNPs) in the structural region of the *MBL2* gene, located in codons 52 (A/D), 54 (A/B), and 57 (A/C) of exon 1, and two promoter polymorphisms, located at − 550H/L and − 221X/Y, are the major determinants of serum MBL concentration and activity^12–14^. The variant B, C and D alleles, collectively referred to as the O allele, are associated with serum MBL deficiency compared to the wild-type A allele^12,13^. In addition, polymorphisms within the promoter region (− 550H/L and − 221X/Y) determine the serum MBL concentration to an extent by influencing gene expression^14^. Host *MBL2* genetic variations are associated with susceptibility to a wide variety of infectious and autoimmune diseases^15^, including tuberculosis (TB)^16^, rheumatoid arthritis^17^, systemic lupus erythematosus (SLE)^18^ and GBS^19^. However, other reported *MBL* polymorphisms protect against infectious disease like TB^20^. MBL can act as double-edged sword in post-infectious diseases as GBS depending on its concentration. Serum MBL deficiency has been reported as a predisposing factor for the development of SLE^21^, atherosclerosis^22^, TB^16^ and coronary artery disease^23^. However, some studies reported elevated serum MBL was associated with increased disease susceptibility^24^, thus, the data remain inconclusive. These inconsistent results may be due to the relatively small study populations, different ethnic groups, and environmental influences between studies.

Host–pathogen interactions and environmental factors have attracted attention as concepts that may contribute to GBS. Identifying GBS susceptibility genes would represent an advancement in our understanding of the pathogenesis of the disease. Previously, we reported immune-mediated genes, including tumor necrosis factor-alpha^25^, apoptotic gene FAS^26^ and toll-like receptor-4^27^ were associated with GBS disease susceptibility, whereas human leukocyte antigen- DQB1^28^, antigen presenting glycoprotein CD1A and CD1E^29^ genes have no effect on development of GBS. However, very limited data are available on *MBL2* gene polymorphisms and their influence on the serum MBL levels in patients with GBS. Therefore, we determined the potential association between *MBL2* gene polymorphisms and disease susceptibility, clinical subtypes, severity and level of serum MBL protein in patients with GBS.

## Results

### Clinical and sociodemographic characteristics

The basic demographic and clinical characteristics of all patients with GBS included in this study are summarized in Table 1. The median age was 30 years (interquartile range [IQR], 18–42 years) for GBS patients (*n* = 300) and 34 years (IQR, 28–46 years) for healthy controls (*n* = 300). Almost 225/300 (75%) patients with GBS had a history of preceding events, mostly diarrhea (129/300; 43%) or respiratory tract infections (54/300; 18%). Electrophysiological studies were performed on 240/300 patients (80%), resulting in classifications of the axonal variant for 143/240 (60%) cases (including acute motor axonal neuropathy [AMAN] for 131/240 [55%] cases and acute motor and sensory axonal neuropathy [AMSAN] for 12/240 [5%] cases); acute inflammatory demyelinating polyradiculoneuropathy (AIDP) for 66/240 (28%) cases; unclassified GBS with unexcitable nerves or equivocal findings for 23 (9%) cases and normal for 8/240 (3%). The majority of patients 230/300 (79%) were severely affected at the time of enrolment on the basis of their Medical Research Council (MRC) sum score; 56/300 (19%) patients had dysautonomia and 44/300 (15%) required mechanical ventilation (MV), 248/300 (83%) patients with GBS regained locomotion and 18/300 (6%) died within six months of follow up.

**Table 1:** Demographic, clinical and serological information of the study patients (*n* = 300).

Characteristics	Number of patients (%)
**Sex**	
Male/ Female	204/96 (68/32)
**Age**	
Median (IQR)	30 (18–42)
**Preceding events**	225 (75)
Diarrhea	129 (43)
Respiratory tract infections	54 (18)
Fever	15 (5)
Other	27 (9)
**Electrophysiological classification (*n* = 240)**	
Axonal (AMAN and AMSAN)	143 (60)
Demyelinating	66 (28)
Unclassified	23 (9)
Normal	8 (3)
**Serology**	
Anti-GM1-Ab seropositive	115 (38)
*C. jejuni* seropositive	176 (59)
**Severity based on MRC sum score (at entry)**	
Severely affected (MRC < 40)	230 (79)
Mildly affected (MRC 40–60)	70 (21)
**Clinical outcome of GBS at 6 months**	
Able to walk independently	248 (83)
Unable to walk	34 (11)
Died	18 (6)

*IQR* interquartile range, *Ab* antibody, *C. jejuni Campylobacter jejuni.*

### No association between *MBL2* gene polymorphisms and GBS susceptibility

The distributions of *MBL2* genotypes were compared between patients with GBS and healthy controls to assess the association with GBS susceptibility. All genotype distributions in healthy controls fitted Hardy–Weinberg equilibrium. There was no significant difference in the genotype and allele frequencies between patients with GBS and controls. In particular, genotypes and alleles of − 550 (H/L), − 221 (X/Y) and exon 1 (A/O) SNPs occurred at similar frequencies in patients with GBS and healthy controls (Table 2). In addition to single-site SNP analysis, we compared the haplotypes of the patients with GBS and controls. The haplotypes were determined by combining the following alleles: − 550H/L and − 221X/Y (HY haplotypes); − 550H/L and exon 1A/O (HA haplotype); − 221X/Y and exon 1 A/O (YA haplotypes) and − 550H/L, − 221X/Y and exon 1A/O (HYA haplotypes). The HY haplotype contains heterozygous combination of HY (HY/LX & HY/LY) and homozygous combination of HY (HY/HY) and no combination of HY (LX/LX, LX/LY & LY/LY) genotypes. The distribution of the HY, HA, YA and HYA haplotypes were almost similar between cases and controls (Table 2).

**Table 2:** Associations of *MBL2* polymorphisms among patients with GBS and healthy controls.

SNPs	Genotypes, alleles and haplotypes	GBS patients, *n* = 300, (%)	Healthy controls, *n* = 300, (%)	*P*-value	OR (95% CI)
− 550 (H/L)	LL	120 (40)	123 (41)	Reference	
	HL	142 (47)	144 (48)	1.00	1.01 (0.72–1.42)
	HH	38 (13)	33 (11)	0.59	1.18 (0.69–2.00)
	L Allele	382 (64)	390 (65)	Reference	
	H Allele	218 (36)	210 (35)	0.67	1.06 (0.84–1.34)
− 221 (X/Y)	YY	178 (59)	169 (56)	Reference	
	XY	99 (33)	114 (38)	0.30	0.82 (0.59–1.16)
	XX	23 (8)	17 (6)	0.51	1.29 (0.66–2.49)
	Y Allele	455 (76)	452 (75)	Reference	
	X Allele	145 (24)	148 (25)	0.89	0.97 (0.75–1.27)
Exon 1 (A/O)	AA	178 (59)	177 (59)	Reference	
	AO	105 (35)	110 (37)	0.80	0.95 (0.68–1.33)
	OO	17 (6)	13 (4)	0.57	1.30 (0.61–2.76)
	A Alleles	461 (77)	464 (77)	Reference	
	O Alleles	139 (23)	136 (23)	0.89	1.03 (0.79–1.35)
HY haplotype	No HY allele	121 (40)	123 (41)	Reference	
	HY heterozygous	141 (47)	144(48)	1.00	1.00 (0.71–1.40)
	HY homozygous	38 (13)	33 (11)	0.59	1.17 (0.69–1.99)
HA haplotype	No HA allele	127 (42)	129 (43)	Reference	
	HA heterozygous	150 (50)	152 (51)	1.00	1.00 (0.72–1.40)
	HA homozygous	23 (8)	19 (6)	0.62	1.23 (0.64–2.37)
YA haplotype	No YA allele	40 (13)	30 (10)	Reference	
	YA heterozygous	177 (59)	191 (64)	0.19	0.70 (0.42–1.16)
	YA homozygous	83 (28)	79 (26)	0.47	0.79 (0.45–1.39)
HYA haplotype	No HYA-allele	128 (43)	128 (43)	Reference	
	HYA heterozygous	148 (49)	153 (51)	0.87	0.97 (0.69–1.35)
	HYA homozygous	24 (8)	19 (6)	0.51	1.26 (0.66–2.42)

The Bonferroni-adjusted significance threshold was 0.0167 for genotypes and 0.025 for alleles.

*SNPs* single nucleotide polymorphisms, *GBS* Guillain Barré syndrome, *P-value* probability value, *OR* odds ratio, *95% CI* 95% confidence interval.

### The − 550H/L SNP and *MBL2* haplotypes are associated with a less severe form of GBS

The patients enrolled in this study were classified based on their disease severity, as severely affected (MRC sum score of < 40) or mildly affected (MRC sum score between 40 and 60). The frequency of the *MBL2* genotypes were compared among these groups to determine the effect of SNPs on disease severity. The heterozygous HL genotype (63% vs. 43%; *P* = 0.0145, OR = 2.1, 95% CI 1.17–3.82, corrected; *P*c = 0.0048, Table 3) was significantly more frequent in mildly affected patients than severely affected patients with GBS. The heterozygous XY genotype (39% vs. 31%) and heterozygous AO genotype (41% vs. 33%) were also more frequent among mildly affected patients than patients with the severe form of GBS, but these trends were not statistically significant (Table 3).

**Table 3:** Associations between *MBL2* SNPs and GBS disease severity.

Genotypes, alleles and haplotypes	Severely affected, *n* = 230, (%)	Mildly affected, *n* = 70, (%)	*P*-value	OR (95% CI)	Bonferroni adjustment (*Pc*)
**Promoter region − 550 (H/L)**					
LL	99 (43)	21 (30)	Reference		
HL	98 (43)	44 (63)	0.0145	2.1 (1.17–3.82)	*0.0048
HH	33 (14)	5 (7)	0.623	0.71 (0.25–2.05)	0.2076
H-Allele	164 (36)	54 (39)	Reference		
L-Allele	296 (64)	86 (61)	0.548	0.88 (0.60–1.30)	0.274
**Promoter region − 221 (X/Y)**					
YY	137 (60)	41 (59)	Reference		
XY	72 (31)	27 (39)	0.468	1.25 (0.71–2.20)	0.156
XX	21 (9)	2 (3)	0.175	0.32 (0.07–1.42)	0.0583
Y-Allele	346 (75)	109 (78)	Reference		
X-Allele	114 (25)	31 (22)	0.574	0.86 (0.55–1.36)	0.287
**Exon 1 (A/O)**					
AA	138 (60)	40 (57)	Reference		
AO	76 (33)	29 (41)	0.39	1.32 (0.75–2.29)	0.13
OO	16 (7)	1 (2)	0.196	0.22 (0.03–1.68)	0.0653
A-Allele	352 (77)	109 (78)	Reference		
O-Allele	108 (23)	31 (22)	0.819	0.93 (0.59–1.46)	0.4095
**HY haplotype**					
No HY Allele	102 (44.4)	21 (30)	Reference		
HY Heterozygous	95 (41.3)	44 (63)	0.0067	2.25 (1.25–4.06)	*0.0022
HY Homozygous	33 (14.3)	5 (7)	0.801	0.73 (0.26–2.11)	0.267
**HA haplotype**					
No HA Allele	106 (46.1)	21 (30)	Reference		
HA Heterozygous	105 (45.6)	47 (67)	0.0075	2.26 (1.26–4.04)	*0.0025
HA Homozygous	19 (8.3)	2 (3)	0.62	0.53 (0.12–2.46)	0.2067
**YA haplotype**					
No YA Allele	37 (16.1)	3 (4)	Reference		
YA Heterozygous	125 (54.3)	51 (73)	0.0086	5.03 (1.48–17.1)	*0.0029
YA Homozygous	68 (29.6)	16 (23)	0.161	2.9 (0.79–10.6)	0.0537
**HYA haplotype**					
No HYA Allele	108 (47.0)	21 (30)	Reference		
HYA Heterozygous	101 (43.9)	47 (67)	0.0032	2.39 (1.34–4.28)	*0.0011
HYA Homozygous	21 (9.1)	2 (3)	0.536,	0.49 (0.11–2.25)	0.1787

*SNPs* single nucleotide polymorphisms, *GBS* Guillain Barré syndrome, *P-value* probability value, *OR* odds ratio, *95% CI* 95% confidence interval.

The Bonferroni-adjusted significance threshold was 0.0167 for genotypes and 0.025 for alleles, *statistically significant after *P* value correction.

Haplotype analysis of the *MBL2* gene polymorphisms revealed a significant association with developing the mild form of GBS. The HYA heterozygous haplotype (HYA/LYO, HYA/LYA, HYA/LXA, HYA/HYO and HYA/LXO) was significantly more prevalent among mildly affected patients (67%) than severely affected patients (44%) (*P* = 0.0032, OR = 2.39, 95% CI 1.34–4.28, *P*c = 0.0011; Table 3). In addition, the heterozygous HY (*P* = 0.0067, OR = 2.25, 95% CI 1.25–4.06; *P*c = 0.0022), heterozygous YA (*P* = 0.0086, OR = 5.03, 95% CI 1.48–17.1; *P*c = 0.0029) and heterozygous HA (*P* = 0.0075, OR = 2.26, 95% CI 1.26–4.04; *P*c = 0.0025) haplotypes were significantly more prevalent in patients with the mild form of GBS (Table 3).

We also compared the *MBL2* polymorphisms with other severe clinical features like dysautonomia and requirement of mechanical ventilation in patients with GBS. Higher HL and lower LL genotypes were significantly present in patients with dysautonomia than patients with normal autonomic function (*P*^*a* ^= 0.017, OR = 2.29, 95% CI 1.18–4.44, Table 4). Moreover, AO genotype was also significantly associated with autonomic dysfunction (*P*^*a*^ = 0.032, OR = 1.92, 95% CI 1.06–3.47, Table 4). No significant association was found between *MBL2* gene polymorphism and requirement of mechanical ventilation (Table 4). HL and OO genotype were higher among patients who died during the course of the disease.

**Table 4:** *MBL2* gene polymorphism association with autonomic dysfunction and mechanical ventilation in GBS.

Genotypes	Dysautonomia	No-dysautonomia	*P*^*a*^; OR (95% CI)	MV	Non-MV	*P*^*b*^; OR (95% CI)
	*n* = 56 (%)	*n* = 244 (%)		*n* = 44 (%)	*n* = 256 (%)	
**Promoter region − 550H/L**						
LL	15 (27)	105 (43)	Reference	15 (34)	105 (41)	Reference
HL	35 (62)	107 (44)	0.017*; 2.29 (1.18–4.44)	25 (57)	117 (46)	0.302; 1.49 (0.74–2.99)
HH	6 (11)	32 (13)	0.784; 1.31 (0.47–3.66)	4 (9)	34 (13)	0.788; 0.82 (0.26–2.65)
**Promoter region − 221X/Y**						
YY	34 (61)	144 (59)	Reference	28 (64)	150 (59)	Reference
XY	17 (30)	82 (34)	0.748; 0.88 (0.46–1.67)	14 (32)	85 (33)	0.732; 0.88 (0.44–1.77)
XX	5 (9)	18 (7)	0.781; 1.18 (0.41–3.39)	2 (4)	21 (8)	0.539; 0.51 (0.11–2.30)
**Exon 1A/O**						
AA	28 (50)	150 (62)	Reference	25 (57)	153 (60)	Reference
AO	28 (50)	78 (32)	0.032*; 1.92 (1.06–3.47)	17 (39)	88 (34)	0.729; 1.18 (0.61–2.31)
OO	0 (0)	16 (6)	–	2 (4)	15 (6)	1.00; 0.82 (0.18–3.79)

*P*^*a*^-value probability value of dysautonomia vs. no-dysautonomia, *OR* odds ratio, *95% CI* 95% confidence interval, *MV* required mechanical ventilation, *Non-MV* not required mechanical ventilation.

*P*^*b*^-value probability value of MV vs. non-MV.

### Association of *MBL2* polymorphisms with the clinical and serological features of GBS

We categorized the patients with GBS based on electrophysiological subtypes (axonal and demyelinating) and serological data to assess the associations between these features and *MBL2* genotypes. No significant association was found with the *MBL2* genotypes and electrophysiological variants (axonal; AMAN & AMSAN and demyelinating; AIDP) compared to the control group (Supplementary Table S1). The comparison of *MBL2* genotypes and haplotypes between anti-GM1 antibody-positive and antibody-negative group did not reveal a significant association between the *MBL2* polymorphisms and anti-GM1 antibody productivity (Supplementary Table S2). Similarly, there was no significant association found between the *MBL2* genotypes and recent *C. jejuni* infection (data not shown).

### Elevated serum MBL level is associated with disease severity

**Figure 1:**
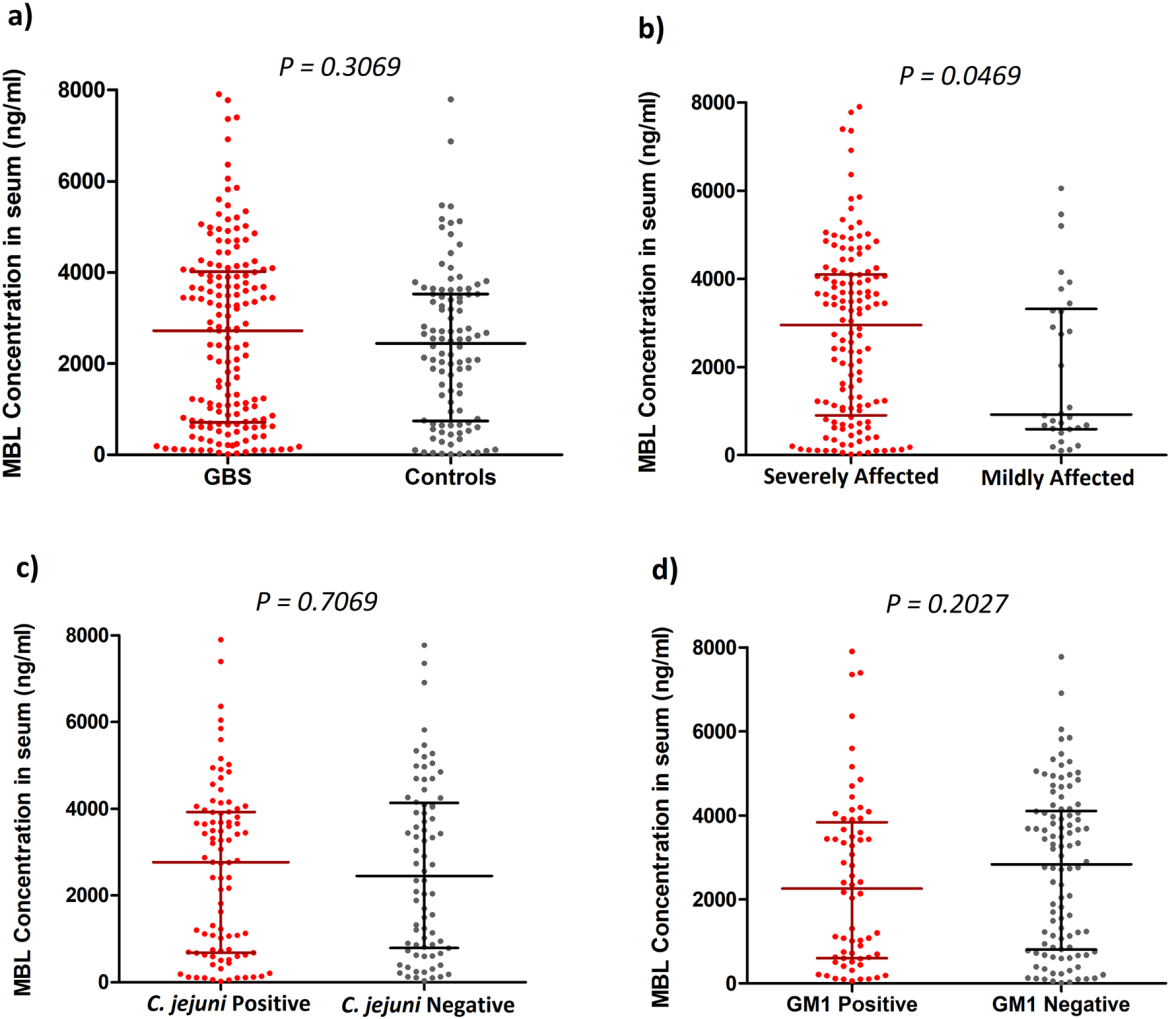
Serum levels of MBL among different subgroups of patients with GBS and healthy controls. Comparisons between (**a**) GBS patients and controls, (**b**) severely affected and mildly affected patients with GBS, (**c**) *C. jejuni*-positive and -negative patients with GBS, and (**d**) anti-GM1 antibody-positive and -negative patients. The thick horizontal black lines indicate the median serum MBL value and the vertical lines indicate the IQR; *P < 0.05, Mann–Whitney *U*-test.

We measured the serum concentration of MBL for a subgroup of patients with GBS (*n* = 166) and healthy controls (*n* = 102). The serum MBL levels were slightly higher in patients with GBS than healthy controls (median, [IQR]; 2726 [715–4013] ng/mL vs. 2440 [738–3524] ng/mL, *P* = 0.3069; Fig. 1a), though serum MBL was not significantly associated with disease susceptibility. The serum concentration of MBL was also compared between the clinical and serological subgroups of GBS. Serum MBL was significantly elevated among severely affected patients compared to mildly affected patients with GBS (median [IQR]; 2957 [903–4093] ng/mL vs. 921 [597–3318] ng/mL, *P* = 0.0469; Fig. 1b). However, we found no significant difference in the serum MBL between the *C. jejuni* or anti-GM1 antibody seropositive and negative groups (Fig. 1c,d). In addition, we excluded the patients with OO genotype and compared the MBL level among the groups. We found high serum MBL level was associated with the severely affected patients with GBS (*P* = 0.0126) and no significant difference was revealed between GBS and healthy control. We compared MBL serum levels with the requirement of mechanical ventilation and development of autonomic, dysfunction in patients with GBS. However, no association was found with the elevated serum MBL and mechanical ventilation or autonomic dysfunction. The median of serum MBL (4417 ng/mL) increased among the patients who died during the course of the disease.

### The − 550H/L and Exon 1A/O SNPs are associated with low levels of serum MBL

**Figure 2:**
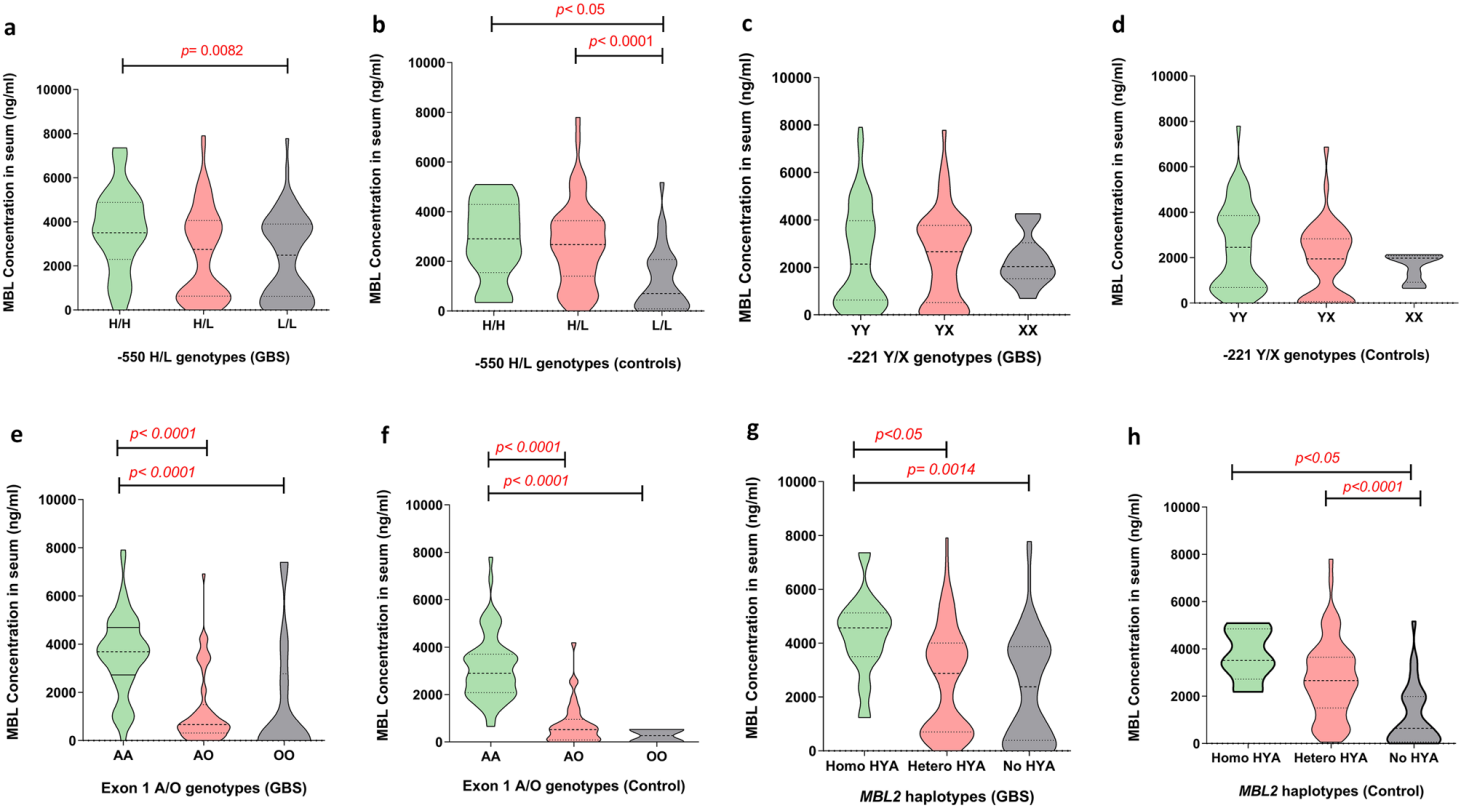
Association of serum MBL levels with *MBL2* genotypes in patients with GBS and healthy controls. (**a–d)** Associations between the promoter − 550H/L and − 221X/Y SNPs and serum MBL levels, (**e,f)** association between functional region exon 1 SNPs and serum MBL levels, and (**g,h)** association between HYA haplotypes and serum MBL levels. The violin plots indicate interquartile (Q3-Q1) ranges with the minimum and maximum values; *P* < 0.05 were determined with Kruskal–Wallis test and the kernel density estimate depicts the multimodal probability distribution.

We compared the serum MBL concentration across the *MBL2* genotypes among the patients with GBS and healthy controls to investigate the influence of *MBL2* polymorphisms on MBL protein levels. In patients with GBS, the homozygous LL promoter genotype was associated with lower serum MBL compared to the HH homozygous genotype (median [IQR]; 1829 [319–3494] ng/mL, vs. 3550 [2295–4881] ng/mL, *P* = 0.0082; Fig. 2a). In healthy controls, serum MBL was significantly lower among individuals with the homozygous LL genotype than individuals with the HH homozygous (median [IQR]; 705, [115–2700] ng/mL vs. 3717 [2577–4295] ng/mL, *P* < 0.05; Fig. 2b) or HL heterozygous genotype (median [IQR]; 705, [115–2700] ng/mL vs. 3193 [2580–3885] ng/mL, *P* < 0.0001; Fig. 2b). The serum MBL concentration was also lower in individuals with the AO heterozygous or OO homozygous genotypes compared to the AA homozygous genotype in both patients with GBS (median [IQR]; 621 [213–1130] ng/mL vs. 3486 [2377–4174] ng/mL, *P* < 0.0001 and 83 [56–729] ng/mL vs. 3486 [2377–4174] ng/mL, *P* < 0.0001; Fig. 2e) and healthy controls (median [IQR]; 595 [108–2426] ng/mL vs. 3520 [2722–4086] ng/mL, *P* < 0.0001 and 479 [432–526] ng/mL vs. 3520 [2722–4086] ng/mL, *P* < 0.0001; Fig. 2f). We found no significant difference in the serum MBL levels among individuals with the − 221X/Y polymorphism (Fig. 2c,d). Comparison between *MBL* haplotypes and serum MBL levels revealed, reduced serum MBL levels were associated with no HYA allele and HYA heterozygous than HYA homozygous in both patients with GBS (*P* = 0.0014 and *P* < 0.05; Fig. 2g) and healthy controls haplotypes (*P* < 0.05 and *P* < 0.0001; Fig. 2h).

## Discussion

Preliminary studies of genetic variation in the *MBL2* gene and its functions over the last decades have focused on disease susceptibility of GBS; however, the roles of *MBL2* in immunopathogenesis and regulation of disease severity are poorly understood. The present study focused on the contribution of polymorphisms in the promoter region (− 550H/L and − 221X/Y) and structural exon 1(A/O) region of the *MBL2* gene and their association with GBS susceptibility, subtype and severity. The analysis of genetic associations in this study revealed *MBL2* gene polymorphisms do not significantly contribute to GBS susceptibility. However, the serum levels of MBL were significantly associated with the HL genotype and heterozygote haplotypes (HY, YA, HA and HYA) of the *MBL2* gene and the disease severity of GBS in our Bangladeshi cohort.

Complement activation has recently been reported to be involved in the induction of post-infectious immune-mediated peripheral nerve damage in patients with GBS^30^. MBL is a key regulatory element that activates the complement system, as part of first-line defense in the pre-immune host in several autoimmune neurological disorders^31^. Previously, Geleijns et al. reported *MBL* polymorphisms contributed to the susceptibility in patients with GBS and were associated with disease severity^19^. The B allele of exon 1 was found to protect against developing severe form of GBS in a Dutch population^19^. However, this study from Bangladesh revealed no significant association between the B allele and disease severity. The probable explanation could be the inclusion of high number of severe cases (77%) in Bangladeshi cohort due to high severity^32^. In contrast, the heterozygous HL, HY, YA, HA and HYA haplotypes were significantly associated with developing the mild form of GBS. In addition, HL and AO genotypes were significantly associated with autonomic dysfunction that includes hypertension, hypotension, tachycardia, pupillary abnormality. However, the previous study did not examine any association between the clinical and serological subgroups, including the subtype of GBS, recent infection with *C. jejuni* and auto-antibodies, with *MBL2* polymorphisms or serum MBL levels^19^. In our population, *MBL2* polymorphisms had no influence on the neural damage that occurs in the axonal and demyelinating variants of GBS. More evidence for relation between MBL and disease susceptibility and severity need to be studied to confirm this finding.

MBL contains pattern recognition features that allow the widest range of potential microbial structures to be recognized^11^. Many infections lead to higher serum MBL levels and increase the disease susceptibility^33,34^. Several reports suggest that the MBL protein can also modulate disease severity including GBS, leptospirosis and dengue^19,34,35^. We observed significant elevation of serum MBL in severely affected patients with GBS, in accordance with the Dutch study^19^. Geleijns et al. reported the only study on GBS and *MBL2* polymorphisms thus far, though, they did not measure serum MBL in their control population which is a limitation of their study^19^. *MBL2* gene polymorphisms appear to influence the serum concentration of MBL as we could confirm both for HC and GBS patients. Three single point mutations in structural codons 52 (A/D), 54 (A/B) and 57 (A/C) of exon 1 of the *MBL2* gene are the major determinants of MBL deficiency^36^. The frequencies of these variant alleles vary in different populations^37,38^. In line with previous observations^19^, we found polymorphism in structural region of exon 1 is associated with lower serum MBL. Individuals with AO and OO genotype had lower serum MBL than the wild-type variant AA homozygous individuals in GBS. The effect of AO and OO genotype on serum MBL in patients with GBS is consistent with the findings in healthy controls, further indicating this polymorphism affects the serum MBL level. Several reports have suggested the promoter region (− 550) variant allele (L allele) downregulates serum MBL by functionally regulating the immune system in several diseases^39^. This study also revealed the L allele was associated with lower serum MBL levels in patients with GBS and the healthy control group.

Several studies have presented evidence that MBL deficiency increases the generalized susceptibility of an individual to infectious diseases^40,41^, including human immunodeficiency virus^42,43^, *Plasmodium falciparum*^44^, cryptosporidium^45^ and *N. meningitidis*^46^. In addition to these associations with infectious disease, there is also evidence of associations between reduced serum MBL and susceptibility to autoimmune diseases. Several reports suggest strong association in the case of systemic lupus erythematosus (SLE). British^47^, Hong Kong Chinese^48^, African American^49^ and Spanish^50^ patients with SLE all exhibited increased frequencies of mutant *MBL* alleles or reduced serum MBL levels. In our Bangladeshi population, we did not find any significant association between GBS disease susceptibility and lower levels of MBL protein. As earlier reported, elevated levels of MBL protein have been associated with increased susceptibility towards chronic rheumatic heart disease in patients previously diagnosed with rheumatic fever^51^. The pathogenesis of rheumatic fever and GBS are similar in terms of antecedent infections, molecular mimicry, and cross-reactive antibodies. Therefore, the higher levels of MBL in severe GBS cases might be explained by binding of MBL to damaged nerve tissue, followed by complement activation, attraction of inflammatory cells, and aggravation of tissue injury. One of the limitations of this study was the lack of determination of complement product activation (C3a and C5a) in serum of GBS patients. Further functional activity of *MBL2* polymorphism and its serum levels can be confirmed by measuring the products of complement pathway.

In conclusion, *MBL2* haplotypes and serum MBL levels may be one key determinant of the severity of weakness in patients with GBS in Bangladesh. Furthermore, both promoter and functional region polymorphisms in the *MBL2* gene are associated with downregulation of serum MBL protein in both patients with GBS and healthy controls. The precise mechanistic role of MBL in GBS is not clear. Further in-depth research is required to elucidate complement activation mediated by MBL and its involvement in the lectin pathway during the pathogenesis of GBS; analysis of samples from the multi-center International GBS Outcome Study (IGOS)^52^ of a large population of various ethnic groups around the world may to explain the underlying mechanisms.

## Materials and methods

### Study participants

The study was conducted in the Laboratory of Gut-Brain Signaling, icddr,b, Dhaka, Bangladesh. The patients with GBS and healthy controls were recruited from Dhaka Medical College and Hospital (DMCH) and the National Institute of Neurosciences and Hospital (NINS), Dhaka, Bangladesh. All patients with GBS fulfilled with the National Institute of Neurological Disorders and Stroke (NINDS) criteria and were enrolled within the two weeks of onset of weakness^53^. The study was reviewed and approved by the Ethical Committee of icddr,b, Dhaka, Bangladesh and all participants gave written informed consent prior enrolment.

Clinical and blood samples were obtained from 300 consecutive patients with GBS and 300 healthy controls for this study. The patients were followed up and clinically assessed at standard time-points (2 weeks, 4 weeks, 6 months and 1 year after entry) to evaluate disease outcome. Disease severity was defined using the Medical Research Council (MRC) sum score for six muscles in the upper and lower limbs on both sides; the sum score ranges from 60 (normal) to 0 (quadriplegic)^54^. GBS patients with a MRC sum score < 40 were defined as severely affected and 40–60, mildly affected^55^. Disease outcome was assessed using the GBS disability scale (GBS-DS)^56^ at six months follow-up; patients able to walk independently (GBS-DS of ≤ 2) were defined as having a good outcome and patients unable to walk independently (GBS-DS of ≥ 3), poor outcome^57^. Ethnicity-based healthy controls (HC) were recruited from people accompanying the patients to physical examinations at the hospitals mentioned above and pair matched with the GBS cases by age and gender. All control subjects were genetically unrelated inhabitants of Bangladesh without a prior history of other neurological complications or non-communicable diseases, and no recent history of pregnancy or surgery.

### Genomic DNA isolation

Genomic DNA was isolated from whole blood for 600 subjects using QIAamp® DNA Blood Midi Kits (100) (Qiagen, Hilden, Germany) following the manufacturer’s instructions. The extracted DNA was dissolved in 1 × TE-buffer (10 mM Tris–Cl, pH 8.0, 1 mM EDTA) and stored at − 80 °C. The stock DNA was diluted to a final concentration of 10 ng/μL in Milli-Q-water and stored at − 20 °C until SNP detection.

### Serological assays

Serological tests were performed on the 300 sera of patients with GBS to assess baseline anti-ganglioside antibodies and recent *C. jejuni* infection at the time of enrolment. Enzyme-linked immunosorbent assay (ELISA) was used to detect IgG, IgM and IgA antibodies against *C. jejuni* and IgG against the nerve GM1-ganglioside, as described previously^58,59^. Serum MBL was measured in duplicate from 166 randomly selected serum samples from patients with GBS at the time of enrolment and 102 serum samples from healthy controls by ELISA method using a human MBL oligomer ELISA kit (BioPotro Diagnostics A/S, Gentofte, Denmark), which specifically detects oligomerized forms of MBL protein. Serum samples were stored at − 80 °C and diluted 1:100 in the sample diluent provided in the ELISA kit during the experiment. The ELISA was performed according to the manufacturer’s instructions; absorbance values were measured at 450 nm and serum concentrations were expressed as nanograms of MBL per milliliter (ng/mL).

### Genotyping of *MBL2* polymorphisms

Single nucleotide polymorphisms (SNPs) in the promoter regions at − 550 (H/L, rs11003125), − 221 (X/Y, rs7096206) and the structural region exon 1 at codon 52 (A/D, rs5030737), codon 54 (A/B, rs1800450) and codon 57 (A/C, rs1800451) of the *MBL2* gene were determined in 600 DNA samples extracted from patients with GBS and healthy controls. The real-time reverse transcription polymerase chain reaction (RT-qPCR) and melting curve analysis were performed in LightCycler capillaries (Roche Diagnostics). The primers, probes and the PCR amplification program were based on Geleijns et al.^19^. The PCR amplification program was designed to determine both promoter regions (− 550H/L and − 221X/Y) and exon 1 (A/O) SNPs using 1 × LightCycler DNA Master Hybridization Probes (Roche Molecular Biochemicals). The melting curve profile was determined after PCR by constant detection of emitted light ^19^. Approximately 20% of the samples were sequenced to confirm the *MBL2* polymorphisms.

### Statistical analysis

The chi-squared test was conducted to examine the differences in demographic characteristics and potential confounders between the GBS cases and healthy controls. The *MBL2* genotype and haplotype frequencies were determined by a simple counting method and managed using Microsoft Excel 2010 (Microsoft, Redmond, WA) and SPSS (16.0 version, Chicago, IL). Fisher's exact test was used to determine the association of *MBL2* polymorphisms and GBS susceptibility; odd ratios (ORs) and 95% confidence intervals (95% CI) were reported. The chi-square test was conducted to confirm the Hardy–Weinberg equilibrium among healthy controls. Serum MBL levels were presented as medians and interquartile ranges (IQR). The non-parametric Mann–Whitney U-test was used to compare the differences in the serum MBL concentrations (ng/mL) between subgroups of patients with GBS and healthy controls. The associations between serum MBL levels and *MBL2* genotypes for the studied SNPs in patients with GBS and controls were analyzed using the Kruskal–Wallis test. The associations between the clinical features of GBS and genotypes were analyzed using Fisher's exact test. A probability level (*P*) of less than or equal 0.05 was considered as the significance criterion. To avoid type I errors in multiple comparisons, the Bonferroni adjustment was performed to correct the *P-*values obtained by dividing the number of comparisons; the corrected *P*-values are denoted as *Pc*^60^. The significance threshold for Fisher's exact test was adjusted to 0.01667 and 0.025 to obtain *Pc* for genotypic and allelic differences. All statistical analyses (except for haplotype analyses) were performed on SPSS software (IBM, Chicago, IL, USA, version 20) and GraphPad Prism (version 5 and 9).

## Supplementary Information

Supplementary Table S1.

Supplementary Table S2.
