# Supplementary material for: Association of mannose-binding lectin 2 gene polymorphisms with Guillain-Barré syndrome
Source: Sci Rep. 2022 Apr 6;12:5791. doi: 10.1038/s41598-022-09621-y (PMC8987049; doi:10.1038/s41598-022-09621-y)
Supplement: Supplementary file 1 — Supplementary Table S1. [file 41598_2022_9621_MOESM1_ESM.docx]

**Supplementary table S1: Association of *MBL2* polymorphism between electrophysiological subtypes and healthy controls.**

|  | **Genotypes, alleles & haplotypes** | **Axonal**  ***n*= 143 (%)** | **Demyelinating**  ***n*= 66 (%)** | **Healthy controls**  ***n*= 300 (%)** | | | **Axonal vs. Control** | **Demyelinating vs. Control** |
| --- | --- | --- | --- | --- | --- | --- | --- | --- |
| ***MBL2* SNPs** |  |  |  |  |  |  | ***P*-value, OR (95%CI)** | ***P*-value, OR (95%CI)** |
| -550 (H/L) | LL | 54 (37.8) | 27 (40.9) | | 123 (41.0) | Reference | | Reference |
|  | HL | 69 (48.2) | 29 (43.9) | | 144 (48.0) | 0.743, 0.92 (0.60-1.41) | | 0.771, 1.09 (0.61-1.94) |
|  | HH | 20 (14.0) | 10 (15.2) | | 33 (11.0) | 0.321, 0.72 (0.38-1.37) | | 0.51, 0.72 (0.32-1.65) |
|  | L- allele | 177 (61.9) | 83 (62.9) | | 390 (65.0) | Reference | | Reference |
|  | H- allele | 109 (38.1) | 49 (37.1) | | 210 (35.0) | 0.370, 0.87 (0.65-1.17) | | 0.688, 0.91 (0.62-1.35) |
| -221 (X/Y) | YY | 88 (61.5) | 36 (54.5) | | 169 (56.3) | Reference | | Reference |
|  | XY | 46 (32.2) | 26 (39.4) | | 114 (38.0) | 0.281, 1.29 (0.84-1.98) | | 0.887, 0.93 (0.53-1.63) |
|  | XX | 9 (6.3) | 4 (6.1) | | 17 (5.7) | 1.00, 0.98 (0.42-2.30) | | 0.771, 0.91 (0.29-2.85) |
|  | Y- allele | 222 (77.9) | 98 (74.2) | | 452 (75.3) | Reference | | Reference |
|  | X- allele | 64 (22.1) | 34 (25.8) | | 148 (24.7) | 0.500, 1.14 (0.81-1.59) | | 0.824, 0.94 (0.61-1.45) |
| Exon 1 (A/O) | AA | 85 (59.4) | 41 (62.1) | | 177 (59.0) | Reference | | Reference |
|  | AO | 47 (32.9) | 22 (33.3) | | 110 (36.7) | 0.664, 1.12 (0.73-1.72) | | 0.668, 1.16 (0.65-2.05) |
|  | OO | 11 (7.7) | 3 (4.5) | | 13 (4.3) | 0.185, 0.57 (0.24-1.32) | | 0.744, 1.00 (0.27-3.69) |
|  | A- allele | 217 (75.9) | 104 (78.8) | | 464 (77.3) | Reference | | Reference |
|  | O- allele | 69 (24.1) | 28 (21.2) | | 136 (22.7) | 0.670, 0.92 (0.66-1.28) | | 0.818, 1.09 (0.69-1.72) |
| HY Haplotypes | No HY allele | 54 (37.8) | 27 (40.9) | | 123 (41.0) | Reference | | Reference |
|  | HY heterozygous | 69 (48.2) | 29 (43.9) | | 144 (48.0) | 0.743, 0.92 (0.60-1.41) | | 0.771, 1.09 (0.61-1.94) |
|  | HY homozygous | 20 (14.0) | 10 (15.2) | | 33 (11.0) | 0.321, 0.72 (0.38-1.38) | | 0.510, 0.72 (0.32-1.65) |
| HA Haplotypes | No HA allele | 59 (41.3) | 29 (43.9) | | 129 (43.0) | Reference | | Reference |
|  | HA heterozygous | 73 (51.0) | 31 (47.0) | | 152 (50.7) | 0.840, 0.95 (0.63-1.44) | | 0.776, 1.10 (0.63-1.93) |
|  | HA homozygous | 11 (7.7) | 6 (9.1) | | 19 (6.3) | 0.674, 0.79 (0.35-1.77) | | 0.584, 0.71 (0.26-1.94) |
| YA Haplotypes | No YA allele | 20 (14.0) | 7 (10.6) | | 30 (10.0) | Reference | | Reference |
|  | YA heterozygous | 83 (58.0) | 41 (62.1) | | 191 (63.7) | 0.188, 1.53 (0.82-2.86) | | 0.820, 1.09 (0.45-2.65) |
|  | YA homozygous | 40 (28.0) | 18 (27.3) | | 79 (26.3) | 0.483, 1.32 (0.67-2.60) | | 1.00, 1.02 (0.39-2.70) |
| HYA Haplotypes | No HYA-allele | 59 (41.3) | 29 (43.9) | | 128 (42.7) | Reference | | Reference |
|  | HYA heterozygous | 73 (51.0) | 31 (47.0) | | 153 (51.0) | 0.915, 0.97 (0.64-1.46) | | 0.776, 1.12 (0.64-1.95) |
|  | HYA homozygous | 11 (7.7) | 6 (9.1) | | 19 (6.3) | 0.674, 0.80 (0.36-1.78) | | 0.585, 0.72 (0.26-1.96) |

SNPs single nucleotide polymorphisms; HC healthy controls; *P*-value probability-value; OR odds ratio; 95% CI 95% confidence interval.
