# Supplementary material for: Association of mannose-binding lectin 2 gene polymorphisms with Guillain-Barré syndrome
Source: Sci Rep. 2022 Apr 6;12:5791. doi: 10.1038/s41598-022-09621-y (PMC8987049; doi:10.1038/s41598-022-09621-y)
Supplement: Supplementary file 2 — Supplementary Table S2. [file 41598_2022_9621_MOESM2_ESM.docx]

**Supplementary table S2: Comparison of *MBL2* SNPs between anti-GM1 antibodies positive and negative patients with GBS.**

| ***MBL2***  **SNPs** | **Genotypes, alleles & haplotypes** | **anti-GM1 antibody** | | ***P*-value** | **OR (95%CI)** |
| --- | --- | --- | --- | --- | --- |
|  |  | **Positive**  ***n*= 115 (%)** | **Negative**  ***n*= 185 (%)** |  |  |
| -550 (H/L) | HH | 42 (36.5) | 78 (42.2) | Reference | Reference |
|  | HL | 57 (49.6) | 85 (45.9) | 0.443 | 0.80 (0.48-1.33) |
|  | LL | 16 (13.9) | 22 (11.9) | 0.445 | 0.74 (0.35-1.56) |
|  | H-Allele | 89 (38.7) | 129 (34.9) | Reference | Reference |
|  | L-Allele | 141 (61.3) | 241 (65.1) | 0.382 | 1.18 (0.84-1.66) |
| -221 (X/Y) | YY | 67 (58.3) | 111 (60.0) | Reference | Reference |
|  | XY | 42 (36.5) | 57 (30.8) | 0.444 | 0.82 (0.49-1.35) |
|  | XX | 6 (5.2) | 17 (9.2) | 0.359 | 1.71 (0.64-4.55) |
|  | Y-Allele | 176 (76.5) | 279 (75.4) | Reference | Reference |
|  | X-Allele | 54 (23.5) | 91 (24.6) | 0.77 | 1.06 (0.72-1.56) |
| Exon 1 (A/O) | AA | 62 (53.9) | 116 (62.7) | Reference | Reference |
|  | AO | 45 (39.1) | 60 (32.4) | 0.205 | 0.71 (0.43-1.17) |
|  | OO | 8 (7.0) | 9 (4.9) | 0.427 | 0.60 (0.22-1.64) |
|  | A-Allele | 169 (73.5) | 292 (78.9) | Reference | Reference |
|  | O-Allele | 61 (26.5) | 78 (21.1) | 0.136 | 0.74 (0.50-1.09) |
| HY Haplotype | No HY Haplotype | 43 (37.4) | 78 (42.2) | Reference | Reference |
|  | HY Heterozygous | 56 (48.7) | 85(45.9) | 0.524 | 0.84 (0.51-1.38) |
|  | HY Homozygous | 16 (13.9) | 22(11.9) | 0.564 | 0.76 (0.36-1.59) |
| HA Haplotype | No HA Haplotype | 46 (40) | 81(43.8) | Reference | Reference |
|  | HA Heterozygous | 59 (51.3) | 91(49.2) | 0.620 | 0.88 (0.54-1.43) |
|  | HA Homozygous | 10 (8.7) | 13 (7.0) | 0.64 | 0.74 (0.30-1.82) |
| YA Haplotype | No YA Haplotype | 13 (11.3) | 26 (14.0) | Reference | Reference |
|  | YA Heterozygous | 73 (63.5) | 103(55.7) | 0.373 | 0.71 (0.34-1.46) |
|  | YA Homozygous | 29 (25.2) | 56(30.3) | 1.0 | 0.96 (0.43-2.12) |
| HYA Haplotype | No HYA Haplotype | 47 (40.9) | 82 (44.3) | Reference | Reference |
|  | HYA Heterozygous | 59 (51.3) | 89 (48.1) | 0.620 | 0.86 (0.53-1.41) |
|  | HYA Homozygous | 9 (7.8) | 14 (7.6) | 0.818 | 0.89 (0.36-2.22) |

SNPs single nucleotide polymorphisms; *P*-value probability-value; OR odds ratio; 95% CI 95% confidence interval.
